# Perioperative Management of Type 2N Von Willebrand's Disease with Recombinant Factor VIII in a Patient Undergoing Knee-Replacement Surgery

**DOI:** 10.1155/2013/837906

**Published:** 2013-02-03

**Authors:** Srivasavi Dukka, David John Allsup

**Affiliations:** Department of Haematology, Queen's Centre for Oncology and Haematology, Castle Hill Hospital, Castle Road, Cottingham, East Riding of Yorkshire HU16 5JQ, UK

## Abstract

Type 2N Von Willebrand's disease (2N VWD) is a rare, recessively inherited bleeding disorder, comprising 1-2% of all VWD patients, usually manifesting as a mild bleeding diathesis. Treatment includes desmopressin (DDAVP) or intermediate purity plasma-derived FVIII concentrates containing residual VWF. We present a case of a 75-year-old gentleman, incidentally diagnosed with type 2N VWD in 2002 on routine blood testing before surgery with an ISTH bleeding score of 1-2, who was treated with recombinant FVIII preoperatively.

Type 2N Von Willebrand's disease (2N VWD) is a rare recessively inherited bleeding disorder, comprising 1-2% of all VWD patients, usually manifesting as a mild bleeding diathesis, although severe cases have been reported. Such patients have disproportionately low factor VIII (FVIII) level secondary to reduced factor VIII binding to Von Willebrand factor (VWF), normal VWF, Ristocetin cofactor activity (RiCof), and multimeric analysis. The R854Q (arginine to glutamine at position 854) mutation in VWF gene is found in just under half of these patients [1]. Treatment includes desmopressin (DDAVP) or intermediate purity plasma-derived FVIII concentrates containing residual VWF.

We present a case of a 75-year-old gentleman, incidentally diagnosed with type 2N VWD in 2002 on routine blood testing before surgery with an ISTH bleeding score of 1-2, who was treated with recombinant FVIII preoperatively.

The patient had no significant bleeding history except for easy bruising and had undergone multiple surgeries uneventfully prior to diagnosis. His past medical history was significant for myocardial infarction with recurrent angina, hypertension, hypercholesterolemia, asthma, sigmoid diverticulosis, acid peptic disease, and osteoarthritis. He previously had carpal tunnel decompressions bilaterally, multiple release operations for trigger fingers, and three surgeries for intervertebral disc prolapse and lumbar canal stenosis. There was no significant family history of a bleeding diathesis.

Investigations prior to his third spinal exploratory surgery and laminectomy revealed a prolonged activated prothromboplastin time (APTT) at 50.4 sec (22–34 sec), normal prothrombin time (PT), thrombin time (TT), and fibrinogen. Further investigations demonstrated a low FVIII of 28.5 IU/lit (40–200 IU/dL), normal VWF, RiCof, and a negative lupus anticoagulant. VWF/FVIII binding assays confirmed a reduction in FVIII binding. Mutational analysis of his VWF gene revealed a homozygous Arg854Gln missense mutation in exon 20, previously described in association with type 2N VWD [1]. Platelet aggregation to ADP, epinephrine, and collagen were reduced, secondary to concurrent use of NSAIDs. He underwent the procedure uneventfully without any factor cover. Since he had ischemic heart disease, he did not undergo a DDAVP response test.

He represented prior to a total knee replacement and in view of the potential for significant blood loss, we elected to offer clotting factor replacement therapy preoperatively. Following a discussion of the theoretical risks of plasma-derived clotting factors with the patient, we opted to cover the surgery with recombinant FVIII, 2000 IU, preoperatively followed by close monitoring of FVIII levels to guide further doses. Thirty minutes following the clotting factor infusion, his FVIII rose to 118 u/L and his levels were maintained at a value not less than 98 u/L without the need for further clotting factor infusions for the entire postoperative course (Table 1).

The surgery was uneventful. Postoperatively, he had a local wound infection but reported no untoward bleeding and did not require blood transfusion.

Apart from mediating platelet adhesion, VWF functions as a carrier protein for FVIII. In type 2N VWD, mutations in the FVIII binding domain of VWF reduce the half life of FVIII and lead to a phenotype of FVIII deficiency. Plasma levels of VIII correlate with the type of mutations. The R854Q mutation has levels of 21.8 ± 9.8 IU/dL and usually tend to have a good, sustained response to DDAVP [2] while more severe cases have FVIII levels of <10 IU/dL with a poor response to DDAVP. The diagnosis of type 2N VWD is confirmed by low FVIII levels, reduced FVIII: VWF binding and confirmed by genetic tests to look for mutations in exons 18–24 [3].

Treatment of VWD type 2N is with DDAVP in patients who respond and have no contraindications to desmopressin (hypersensitivity and cardiac disease). Other patients require plasma-derived VWF concentrates. Rare anecdotal reports exist for the use of recombinant FVIII [4], although these are not recommended based on pharmacokinetic data [5] which suggests a lack of significant, sustained, and durable FVIII levels with concentrates containing only FVIII. However, the risks of infection with plasma products, with agents such as variant CJD, parvovirus, hepatitis A, and possible new agents, are of concern, despite advances in purification techniques virtually eliminating HIV and hepatitis B and C.

Our patient with a mild bleeding history had never been exposed to blood products and had contraindications to DDAVP. Considering his mild bleeding phenotype, it was deemed appropriate to give him a recombinant FVIII prior to surgery and supplement with further doses as required by regular monitoring of his FVIII levels. The postoperative FVIII levels remained haemostatic with no evidence of bleeding.

This case highlights that recombinant FVIII may be a suitable alternative to plasma products in type 2N VWD patients with a low bleeding score and contraindications to desmopressin. We hypothesize that the recombinant FVIII preoperatively raised the levels to 100% and, thus, covered the perioperative period while the endogenous release of FVIII, due to the surgical stress, maintained enough FVIII levels to maintain haemostasis.

## Figures and Tables

**Table 1:** Perioperative APTT and FVIII levels.

	Baseline	After 30 min RFVIII (2000U)	1 hr later	6 hrs after	D + 1	D + 2	D + 3
APTT (22–34 sec)	48.1	38.6	38.5	38.1	37.9	40.7	40.2
FVIII (40–200 IU/dL)	66.3	118.1	138.3	110.7	98	108.9	163.4

D + (number): (number) of days after surgery.
